# Supplementary material for: Improvement in gait stability in older adults after ten sessions of standing balance training
Source: PLoS One. 2022 Jul 27;17(7):e0242115. doi: 10.1371/journal.pone.0242115 (PMC9328559; doi:10.1371/journal.pone.0242115)
Supplement: S1 File — (DOCX) [file pone.0242115.s001.docx]

## **Supplementary material;**

Supplementary material 1

Participants first trained for 30 minutes individually between pre and post1 measurement timepoint on a single day. Then, for three weeks with a frequency of three times per week, for 45 minutes per session, they trained in a group of 4 to 8 people. A training session consisted of blocks of 40-60 second exercises in which balance was challenged by different surface conditions, static conditions, perturbations, and dual tasks. Over the course of the training period, the difficulty level was increased by using more challenging exercises, challenging surface conditions (foam, balance boards), and perturbations. Progression criteria were based on the researcher’s observation during the training sessions; if participants were able to perform the task for 60 seconds, the difficulty level would be increased using different balance boards or by limiting the sensory inputs (S1 Fig). Participants were encouraged to train at their individual balance ability level. To test the transfer of acquired skill to gait, none of the exercises included stepping, jumping, or locomotion. To maintain safety, exercises were carried out in groups of two under supervision of the researchers.

*
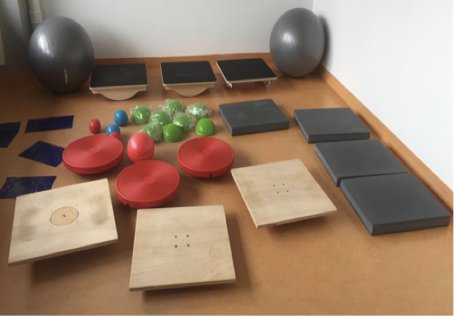
*

*S1 Fig. Balance training materials.*

The progression plan was as follows:

*S1 Table. Guideline for training progression*

| **Number** | **Exercise** | **Duration/Frequency** |
| --- | --- | --- |
| **Warm-up** | | |
| **1** | **head rotations** | **rotate head to either side 5 x**  **3 repetitions** |
| **2** | **back stretching** | **stretch 3 x**  **3 repetitions** |
| **3** | **trunk rotations** | **5 rotations to both sides**  **3 repetitions** |
| **Exercises** | | |
| **4** | **balancing**  **- one leg stance (when possible)**  **- switch the legs**  **- unstable surfaces** | **3 x 60 seconds**  **2 repetitions** |
| **5** | **balancing eyes-closed**  **- one leg stance (when possible)**  **- switch the legs**  **- unstable surfaces** | **3 x 60 seconds**  **2 repetitions** |
| **6** | **displacement of weight**  **- one leg stance**  **- switch the legs**  **- unstable surfaces** | **3 x 60 seconds**  **2 repetitions** |
| **7** | **passing/throwing around a ball in groups of 4**  **fitness ball**  **- one leg**  **- unstable surface**  **2 kg ball**  **- one leg**  **- unstable surface**    **alternative approaches:**  **- make the circle bigger.**  **- with back towards each other in order to induce more trunk rotations.** | **5 rounds both directions**  **3 repetitions** |
| **8** | **pass the big ball around while stopping it on foot and role it to the other person.**  **fitness ball**  **- one leg**  **- unstable surface**  **2 kg ball**  **- one leg**  **- unstable surface** | **5 rounds both directions**  **3 repetitions** |

Supplementary material 2; FES-I, Concern of falls

Several studies showed a strong correlation between concern of falling and balance performance [1,2]. It has been shown that poor balance performance is mediated by changes in the allocation of attention in the presence of concern of falling [1]. Concern of falling is reduced after training in older adults, which is associated with improved balance performance [2,3]. To assess concern of falling, we used the Falls Efficacy Scale International (FES-I) questionnaire at pre, post2, and retention time-points [4]. FES-I outcomes are on a scale of 16 to 64, with 16 indicating minimum concern of falling and 64 severe concern of falling.

A repeated measures ANOVA indicated that concern of falling was affected by balance training (F2,42= 4.37, P = 0.039; S2 Fig.). Post-hoc analysis showed that concern of falling was not significantly changed immediately after the training program, but was decreased at retention (t = 2.16, p = 0.072; t = 2.82, p = 0.022, respectively), implying that weeks after training participants felt more confident about their balance ability.

S2 Fig. FES-I scores at different time points. Each of the lines between timepoints represents the score of a single participant.

References:

1. Young WR, Mark Williams A. How fear of falling can increase fall-risk in older adults: Applying psychological theory to practical observations. Gait Posture. 2015;41: 7–12. doi:10.1016/j.gaitpost.2014.09.006

2. Thiamwong L, Suwanno J. Effects of simple balance training on balance performance and fear of falling in rural older adults. Int J Gerontol. 2014;8: 143–146. doi:10.1016/j.ijge.2013.08.011

3. Kumar A, Delbaere K, Zijlstra GAR, Carpenter H, Iliffe S, Masud T, et al. Exercise for reducing fear of falling in older people living in the community: Cochrane systematic review and Meta-Analysis. Age Ageing. 2016;45: 345–352. doi:10.1093/ageing/afw036

4. Kempen GIJM, Zijlstra GAR, van Haastregt JCM. [The assessment of fear of falling with the Falls Efficacy Scale-International (FES-I). Development and psychometric properties in Dutch elderly]. Tijdschr Gerontol Geriatr. 2007;38: 204–212.
