# Supplementary material for: Improvement in gait stability in older adults after ten sessions of standing balance training
Source: PLoS One. 2022 Jul 27;17(7):e0242115. doi: 10.1371/journal.pone.0242115 (PMC9328559; doi:10.1371/journal.pone.0242115)
Supplement: Attachment — Submitted filename: Response to Reviewers.docx [file pone.0242115.s007.docx]

We would like to thank the reviewers for their valuable comments. Without their comments the article would have not reached this level of quality. Below there are point-by-point answers to reviewers’ comments.

Reviewer #1: The changes based on the previous review have strengthened the manuscript. However, there are a few issues to be addressed to increase clarity and readability.

Major issues:

1. Justification for step width and step width variability based on stability/control of gait. While we (review team) suggested the authors examine this measure, the authors need to provide strong justifications for why these variables were included and add appropriate hypotheses.

Line 107 and lines 122-126 are redundant (“The two latter variables were added to the analysis upon a reviewer’s suggestion” line 107). I appreciate that the authors clearly and explicitly state that SW and SWvar were not initially included. I recommend removing the statement from line 107 and keeping lines 122-126.

However, you should not rely only on the suggestion of a reviewer, there must be a rationale beyond suggestion of a third party. Increase the justification for these measures based on stability/empirical data. You state earlier that SW and SWvar are associated with age and with older fallers (lines 65-67), expand on that.

Thank you for the suggestion. Line 107 has been removed. The texts below have been added to the introduction:

(Lines 67-74): Mediolateral gait stability is thought to be actively controlled by adjusting foot placement (Bruijn & Van Dieën, 2018), as centre of mass kinematics during the preceding swing phase strongly correlate with foot position in the next stance phase (Hurt et al., 2010; Wang & Srinivasan, 2014). The strong coupling between centre of mass kinematics and foot placement is found to decrease in conditions in which gait is stabilized, such as in external lateral stabilization, increasing confidence that lateral gait stability is indeed controlled by foot placement adjustment (Mahaki et al., 2019). This decoupling coincides with a decrease in step width and step width variability (Donelan et al., 2004). Moreover, increased step width and step width variability have been found in older compared to young adults and in fallers compared to non-fallers (Callisaya et al., 2010; Nordin et al., 2010; Skiadopoulos et al., 2020).

(Lines 76-78): Consequently, step width and step width variability are considered to be proxies of the quality of active mediolateral control of gait stability using foot placement adjustments and may therefore be useful to evaluate the effect of balance training.

(Lines 135-146): Even though step width and step width variability seem to be sensitive to quality of balance control in gait, step width measures are not necessarily affected by postural balance training. Postural balance is controlled using torques around the ankle and hip of the standing leg (Nashner, 1985; Runge et al., 1999), but does not include stepping (i.e. foot placement). However, as in postural balance, the stance leg is used to regulate stabilizing torques in the stance phase gait (Reimann et al., 2018). This stance leg control co-determines the control of foot placement (van Leeuwen et al., 2021) and therefore reduced step width and step width variability are likely results of improved control by the stance leg. These predictions on step width and step width variability were conceived, based on reviewer comments, after the data were processed and analyzed. These predictions are thus explorative and aim to drive future research (Kerr, 1998; Rowbottom & Alexander, 2012) and we ask the reader to consider the limitations of the evidence provided by these variables.

2. Skill transfer. Justify the use of the terminology ‘skill transfer’. Step width and step width variability are, of course, not observed during standing, so explicitly state the skill that is transferred? What you observed is a change in gait parameters after standing balance training. This may or may not be ‘skill transfer’, so clarify what skill was transferred and the rationale/evidence that support this interpretation.

We thank the reviewer for the comment. We agree that strictly speaking we do not provide evidence for transfer of acquired skill, while we do provide evidence for transfer of an effect of training. We now use the more neutral term transfer, omitting skill. There are changes throughout the manuscript in Key words, introduction, and discussion.

Lines 37-39: Thus, if effects of standing balance training programs do not transfer to improvements in gait stability, they are unlikely to decrease the number of falls.

Lines 44-46: Consequently, the existence of transfer from standing balance training to gait, as well as the mechanisms underlying such a transfer, if present, are insufficiently clear.

Lines 109-113: In the current study, we aimed to investigate the transfer of effects of standing balance training to normal and narrow-base walking in older adults, as well as the adaptation of older adults to narrow-base walking.

Lines 146-148: While this result is in line with our primary hypothesis and indicates transfer of effects of standing balance training to gait, in a strict sense it cannot be considered a planned analysis.

Line 398: We studied whether effects of standing balance training transferred to gait in older adults.

Lines 403-404: We also expected transfer of training effects to be most pronounced in the narrow-base condition.

Lines 415-416: In this study, after ten sessions we found transfer from standing balance training to gait.

Lines 474-475: (to start with stationary exercises and transfer the training effects to walking)

3. Adaptable and plastic. Line 376 - Authors used the set of terms – adaptable (modulated between conditions) and plastic (modified by training) – for the first time in the discussion. These terms increase clarity when describing/interpreting the different  statistical outcomes. Thus, it would be helpful to include and define these terms in the introduction and perhaps build them into the purpose/aim/hypotheses. Similarly, line 384-386 seems to be related to the condition comparison effects, but not training effects. Be explicit. Please also explain if/how these terms relate to motor modules? We also suggest you use the terms in your headings.

Thank you for the comment. We agree these two terms should have been introduced earlier in the introduction. We now introduce adaptable and plastic in the introduction.

Lines 111- 113: The modulation of balance control between two conditions aimed to test adaptability of balance control to environmental constraints and effects of training were studied to analyse the plasticity of balance control.

Lines 48-50: Fall prevention training programs aim to improve balance control employing plastsicity of the neuromuscular system . To prevent falls, one needs to be able to adapt gait when facing environmental challenges, such as when forced to walk with a narrow step width.
Minor issues:

Line 37-38 – for clarity change “…skills acquired by balance training programs…” to “…skills acquired by standing balance training programs…” (clarification added as many balance training programs including gait activities)

Thank you for the comment. Skills acquired by balance training programs is now changed to effects of standing balance training programs as suggested.

Line 43 – similar comment as above – were the four references [4-8] only examining standing balance training, or balance training that went beyond standing tasks?

Studies 4, 5, and 8 refer to standing balance training. Study 6 used gait and 7 a combination of gait and standing and sitting, lying on the floor, etc. The latter two references have thus been removed.

The text now reads as (Lines 41-43): On the other hand, transfer to gait stability from solely standing balance training [4-6] is suggested by improved clinical balance scores, gait parameters, and performance on the timed up and go, and other tests.

Line 48-56- This paragraph is not clear. What is an ‘altered modulation’ in the last sentence – this seems to be a change in gait that has changed (this may be related to the terms adaptable and plastic, and thus may increase clarity by adding the terms to the introduction)? Also, I don’t follow the logic – if skill transfer (from standing to gait) occurs, then you expect gait to be different between normal and narrow-base walking (NW and NBW, respectively)? Or do you expect a *greater* difference between NW and NBW after training than before training? If the latter is an accurate description of the expectation, why is that expected? Further, the following statement is unclear - “increased confidence after training may result in less adaptation to a challenging condition”. I would assume that increased confidence in a task would allow more adaptations. Also, confidence is included in this paragraph, but is only superficially addressed in one sentence, and is not included in the concluding statement of the paragraph.

Thank you for the comments. Indeed, we expect a change in the difference between NW and NBW after training, that is an interaction between Training and Condition. However, we were unsure about the direction of this effect. We have rephrased this section to (Lines: 49-58):

“To prevent falls, one needs to be able to adapt gait when facing environmental challenges, such as when forced to walk with a narrow step width. Older adults show more pronounced adaptations to narrow-base walking than young adults [11], possibly because they are more cautious in the presence of balance threats [12]. Therefore, an interaction between training and stabilizing demands may be expected. On the one hand, increased confidence after training may result in less adaptation to a challenging condition. On the other hand, balance training may enhance the ability to adapt to challenging conditions. Therefore, if transfer of standing balance training to gait occurs, an altered modulation of gait between normal and narrow-base walking might be expected after training, but the direction of change is unpredictable.”

We expected that training might increase confidence. We did not elaborate on the confidence in the main manuscript, since it was not the focus of the research. However, we provided as short introduction to the topic and the results in the supplementary material for curious readers to test our speculation (copied below).

Several studies showed a strong correlation between concern of falling and balance performance (Thiamwong & Suwanno, 2014; Young & Mark Williams, 2015). It has been shown that poor balance performance is mediated by changes in the allocation of attention in the presence of concern of falling [(Young & Mark Williams, 2015)]. Concern of falling is reduced after training in older adults, which is associated with improved balance performance (Kumar et al., 2016; Thiamwong & Suwanno, 2014). To assess concern of falling, we used the Falls Efficacy Scale International (FES-I) questionnaire at pre, post2, and retention time-points (Kempen et al., 2007). FES-I outcomes are on a scale of 16 to 64, with 16 indicating minimum concern of falling and 64 severe concern of falling.

A repeated measures ANOVA indicated that concern of falling was affected by balance training (F2,42= 4.37, P = 0.039; Figure S2.1). Post-hoc analysis showed that concern of falling was not significantly changed immediately after the training program, but was decreased at retention (t = 2.16, p = 0.072; t = 2.82, p = 0.022, respectively), implying that weeks after training participants felt more confident about their balance ability.

Line 87 – ‘similarly widened activation profiles’ – similar to what?

Thank you for the comment. The word “similarly” has been removed.

Line 134 - Why did authors assume effect size of 0.44. Is it a partial eta squared value?

We chose this effect size from the meta-analysis article, DOI 10.1007/s40279-015-0375-y, which expressed effects size using Cohen’s f. This has now been mentioned in the text as:

Lines 154-158: The required sample size was estimated at twenty-two based on power analysis for an F test of a repeated measures ANOVA, assuming a Cohen’s f of 0.44 (based on meta-analysis of the effect of standing balance training on steady-state balance (Muehlbauer et al., 2015)) and a correlation among repeated measures of 0.6 (β = 0.8, G * power 3.1.9.2, Düsseldorf, Germany), comparable to similar studies (Bisson et al., 2007; Nagy et al., 2007).

Line 158 - specify perturbations (self-perturbation or externally applied, etc.)

We have further specified the perturbations, and the text now reads (Lines 181-182): self-perturbations and external perturbations while catching a ball in a dual tasking exercise.

Line 200- How did authors quantify leg dominance?

We have added this to the methods section as (Lines 225-227): The preferred stance leg was reported by the participant prior to the experiment and confirmed by the experimenter by asking the participant to kick an imaginary soccer ball. The supporting leg was considered the preferred stance leg (Alizadehsaravi et al., 2022).

Line 428 - Authors compared motor modules of novice vs expert ballet dancers and specify that their training period was ten years. It would be helpful to relate how that fits in with the current results.

The mentioned study was a cross-sectional study, in which the experts had a minimum of 10 years training, including all sorts of training in ballet, which could vary from standing balance to gait and more, but with balance control as a core element. The aim of mentioning this study was to show that a long-term training may cause reorganization and permanent changes.

The text (Lines 447-455) now reads as: Even though we found evidence of transfer, gait synergies were not affected by training. Synergy metrics may be insensitive, because subtle variations in the activation of some muscles, for example through improved feedback control, underlie improved balance. Alternatively, changes may have occurred in muscles that we did not include in our measurements. Young adults with years of experience in balance training in ballet showed different motor modules during narrow-base walking compared to novice ballet dancers (Chvatal & Ting, 2013), indicating that long-term effects of training may be apparent in muscle synergies. Note, however, that these ballet dancers’ training experience most certainly included gait-related tasks (Chvatal & Ting, 2013). In addition, the dancers had trained for at least ten years.

Line 451- redundant text with the text in methods section

We removed the redundant text and kept the one in the introduction.

Editorial comments: Line 246 - Underscores before the reference

The underscores have been removed, thank you for noticing that.

Line 291 onwards - At multiple locations, “T” is capitalized for training in the middle of the sentence

Thank you for the comment. We capitalized the T intentionally where we refer to Training as a factor in the ANOVA.

Line 398- Be consistent in format for reporting mean +/- SE 27.6% (SE 3.9%) pre-training and 33.3% (SE 4.4%)

Thanks. We reported the SD now.

Reviewer #2: (No Response)

Reviewer #3: I want to thank the authors for their thoughtful revisions. The new results and the authors’ modifications improved the manuscript considerably. I have just a minor comment. In three parts of the manuscript (two in the Introduction and one in the Discussion), the authors commented on one of the reviewer’s suggestions of adding step width and step width variability variables to argue that these analyses were not pre-planned. You could mention this only in the Discussion (p. 19, lines 451-453). I do not see the reason to keep repeating this information in different parts of the manuscript.

Thank you for the compliment and the time you devoted to review our paper. We have now removed the first statement and acknowledged the changes at the end of the introduction as reviewer #1 suggested and the discussion as you suggested.

References:

Alizadehsaravi, L., Koster, R. A. J., Muijres, W., Maas, H., Bruijn, S. M., & van Dieën, J. H. (2022). The underlying mechanisms of improved balance after one and ten sessions of balance training in older adults. *Human Movement Science*, *81*(December 2021), 1–14. https://doi.org/10.1016/j.humov.2021.102910

Bisson, E., Contant, B., Sveistrup, H., & Lajoie, Y. (2007). Functional balance and dual-task reaction times in older adults are improved by virtual reality and biofeedback training. *Cyberpsychology and Behavior*, *10*(1), 16–23. https://doi.org/10.1089/cpb.2006.9997

Bruijn, S. M., & Van Dieën, J. H. (2018). Control of human gait stability through foot placement. *Journal of the Royal Society Interface*, *15*(143). https://doi.org/10.1098/rsif.2017.0816

Callisaya, M. L., Blizzard, L., Schmidt, M. D., McGinley, J. L., & Srikanth, V. K. (2010). Ageing and gait variability-a population-based study of older people. *Age and Ageing*, *39*(2), 191–197. https://doi.org/10.1093/ageing/afp250

Chvatal, S. A., & Ting, L. H. (2013). Common muscle synergies for balance and walking. *Frontiers in Computational Neuroscience*, *7*(May), 1–14. https://doi.org/10.3389/fncom.2013.00048

Donelan, J. M., Shipman, D. W., Kram, R., & Kuo, A. D. (2004). Mechanical and metabolic requirements for active lateral stabilization in human walking. *Journal of Biomechanics*, *37*(6), 827–835. https://doi.org/10.1016/j.jbiomech.2003.06.002

Hurt, C. P., Rosenblatt, N., Crenshaw, J. R., & Grabiner, M. D. (2010). Variation in trunk kinematics influences variation in step width during treadmill walking by older and younger adults. *Gait and Posture*, *31*(4), 461–464. https://doi.org/10.1016/j.gaitpost.2010.02.001

Kempen, G. I. J. M., Zijlstra, G. A. R., & van Haastregt, J. C. M. (2007). [The assessment of fear of falling with the Falls Efficacy Scale-International (FES-I). Development and psychometric properties in Dutch elderly]. *Tijdschrift voor gerontologie en geriatrie*, *38*(4), 204–212.

Kerr, N. L. (1998). HARKing: Hypothesizing after the results are known. *Personality and Social Psychology Review*, *2*(3), 196–217. https://doi.org/10.1207/s15327957pspr0203_4

Kumar, A., Delbaere, K., Zijlstra, G. A. R., Carpenter, H., Iliffe, S., Masud, T., Skelton, D., Morris, R., & Kendrick, D. (2016). Exercise for reducing fear of falling in older people living in the community: Cochrane systematic review and Meta-Analysis. *Age and Ageing*, *45*(3), 345–352. https://doi.org/10.1093/ageing/afw036

Mahaki, M., Bruijn, S. M., & Van Dieën, J. H. (2019). The effect of external lateral stabilization on the use of foot placement to control mediolateral stability in walking and running. *PeerJ*, *2019*(10). https://doi.org/10.7717/peerj.7939

Muehlbauer, T., Gollhofer, A., Lesinski, M., Hortoba, T., & Granacher, U. (2015). Effects of Balance Training on Balance Performance in Healthy Older Adults : A Systematic Review and Meta-analysis. *Sports Med*, *45*(45), 1721–1738. https://doi.org/10.1007/s40279-015-0375-y

Nagy, E., Feher-Kiss, A., Barnai, M., Domján-Preszner, A., Angyan, L., & Horvath, G. (2007). Postural control in elderly subjects participating in balance training. *European Journal of Applied Physiology*, *100*(1), 97–104. https://doi.org/10.1007/s00421-007-0407-x

Nashner, L. M. (1985). *The organization of human postural movements : A formal basis and experimental synthesis*. *8*, 135–172. https://doi.org/10.1017/S0140525X00020008

Nordin, E., Moe-Nilssen, R., Ramnemark, A., & Lundin-Olsson, L. (2010). Changes in step-width during dual-task walking predicts falls. *Gait & Posture*, *32*(1), 92–97. https://doi.org/https://doi.org/10.1016/j.gaitpost.2010.03.012

Reimann, H., Fettrow, T., & Jeka, J. J. (2018). Strategies for the Control of Balance During Locomotion. *Kinesiology Review*, *7*(1), 18–25. https://doi.org/10.1123/kr.2017-0053

Rowbottom, D. P., & Alexander, R. M. N. (2012). The role of hypotheses in biomechanical research. *Science in Context*, *25*(2), 247–262. https://doi.org/10.1017/S0269889712000051

Runge, C. F., Shupert, C. L., Horak, F. B., & Zajac, F. E. (1999). Ankle and hip postural strategies defined by joint torques. *Gait and Posture*, *10*(2), 161–170. https://doi.org/10.1016/S0966-6362(99)00032-6

Skiadopoulos, A., Moore, E. E., Sayles, H. R., Schmid, K. K., & Stergiou, N. (2020). Step width variability as a discriminator of age-related gait changes. *Journal of NeuroEngineering and Rehabilitation*, *17*(1), 1–13. https://doi.org/10.1186/s12984-020-00671-9

Thiamwong, L., & Suwanno, J. (2014). Effects of simple balance training on balance performance and fear of falling in rural older adults. *International Journal of Gerontology*, *8*(3), 143–146. https://doi.org/10.1016/j.ijge.2013.08.011

van Leeuwen, A. M., van Dieën, J. H., Daffertshofer, A., & Bruijn, S. M. (2021). Ankle muscles drive mediolateral center of pressure control to ensure stable steady state gait. *Scientific Reports*, *11*(1), 1–14. https://doi.org/10.1038/s41598-021-00463-8

Wang, Y., & Srinivasan, M. (2014). Stepping in the direction of the fall: The next foot placement can be predicted from current upper body state in steady-state walking. *Biology Letters*, *10*(9). https://doi.org/10.1098/rsbl.2014.0405

Young, W. R., & Mark Williams, A. (2015). How fear of falling can increase fall-risk in older adults: Applying psychological theory to practical observations. *Gait and Posture*, *41*(1), 7–12. https://doi.org/10.1016/j.gaitpost.2014.09.006
